# The hybrid number of a ploidy profile

**DOI:** 10.1007/s00285-022-01792-6

**Published:** 2022-09-16

**Authors:** K. T. Huber, L. J. Maher

**Affiliations:** University of East Anglia, Norwich, UK

**Keywords:** Phylogenetic network, Ploidy profile, Multiplicity vector, Hybrid number, Simplification sequence, Prime factor decomposition, Binary representation, 05C05, 05C20, 05C85, 05D15, 92D15

## Abstract

Polyploidization, whereby an organism inherits multiple copies of the genome of their parents, is an important evolutionary event that has been observed in plants and animals. One way to study such events is in terms of the ploidy number of the species that make up a dataset of interest. It is therefore natural to ask: How much information about the evolutionary past of the set of species that form a dataset can be gleaned from the ploidy numbers of the species? To help answer this question, we introduce and study the novel concept of a ploidy profile which allows us to formalize it in terms of a multiplicity vector indexed by the species the dataset is comprised of. Using the framework of a phylogenetic network, we present a closed formula for computing the *hybrid number* (i.e. the minimal number of polyploidization events required to explain a ploidy profile) of a large class of ploidy profiles. This formula relies on the construction of a certain phylogenetic network from the simplification sequence of a ploidy profile and the hybrid number of the ploidy profile with which this construction is initialized. Both of them can be computed easily in case the ploidy numbers that make up the ploidy profile are not too large. To help illustrate the applicability of our approach, we apply it to a simplified version of a publicly available Viola dataset.

## Introduction

Datasets such as the Viola dataset considered in Marcussen et al. (2012) arise when species inherit multiple sets of chromosomes from their parents. Generally referred to as polyploidization, this can be due to whole genome duplication (also called autopolyplodization) as in the case of e.g. watermelons and bananas Varoquaux et al. (2000), or by obtaining an additional complete set of chromosomes via hybridization (also called allopolyploidization), as in the case of the frog genus Xenopus Ownbey (1950). This poses the following intriguing question at the center of this paper: How much information about the evolutionary past of a set of species can be gleaned from the *ploidy number* (i.e. the number of complete chromosome sets in a genome) of the species? Evoking parsimony to capture the idea that polyploidization is a relatively rare evolutionary event we re-phrase this question as follows: What is the minimum number of polyploidization events necessary to explain a dataset’s observed *ploidy profile*. For a set *X* of species that make up a dataset, we define such a profile to be the multiplicity vector $(m_1,\ldots , m_n)$ for $n=|X|$, indexed by the species in *X* where, for each $1\le i\le n$, the ploidy number of species $i\in X$ is $m_i\ge 1$.

As it turns out, an answer to this question is well-known if the ploidy profile in question is presented in terms of a multi-labelled tree (see e.g. Huber and Moulton 2006; Huber et al. 2006; Marcussen et al. 2015, 2012). Since it is, however, not always clear how to derive a biologically meaningful multi-labelled tree from the dataset in the first place Huber et al. (2012), we focus here on ploidy profiles for which such a tree is not necessarily available.

**Fig. 1:**
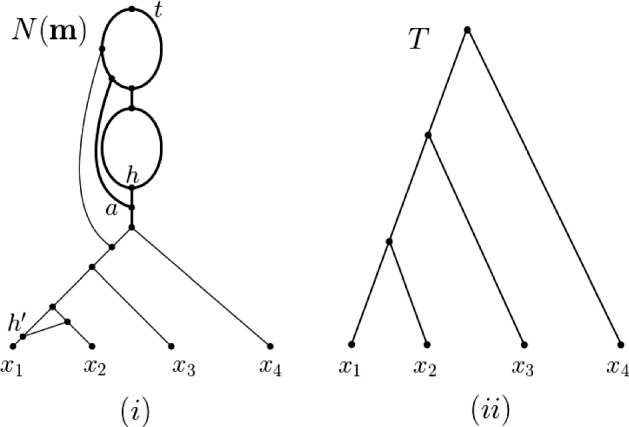
**i** One of potentially many phylogenetic networks that realize the ploidy profile $\mathbf {m}=(12, 6, 6, 5)$ on $X=\{x_1,x_2,x_3,x_4\}$. To improve clarity of exposition, we always assume that arcs are directed downward, away from the root. **ii** A (phylogenetic) tree to which subdivision vertices and arcs have been added to obtain the phylogenetic network in **i** – see the text for details

Due to the reticulate nature of the signal left behind by polyploidization Sagitov et al. (2013), Wagner et al. (2017), Waight et al. (2020), phylogenetic networks offer themselves as a natural framework to formalize and answer our question. Although we present a definition of such structures (and all other concepts used in this section) below, from an intuition development point of view, it suffices to observe at this stage that a phylogenetic network can sometimes be thought of as a rooted directed bifurcating tree *T* with a pre-given set *X* as leaves to which additional arcs have been added via joining subdivision vertices of arcs of *T* so that the following property holds. The resulting graph is a rooted directed acyclic graph with leaf set *X* such that a subdivision vertex *v* of *T* either only has additional arcs starting at it or only additional arcs ending at it. For our purposes we only allow the case that *v* has one additional outgoing arc. Subdivision vertices that have at least one additional incoming arc are called *hybrid vertices* and are assumed to represent reticulate evolutionary events such as polyploidization. If a hybrid vertex in a phylogenetic network *N* also has overall degree three then *N* is generally called a *binary* phylogenetic network. We refer the interested reader to Fig. 1i for an example of a binary phylogenetic network on $X=\{x_1,x_2,x_3,x_4\}$ that is obtained from the tree depicted in Fig. 1ii and to Gusfield (2014), Huber and Moulton (2013), Huson et al. (2010), Steel (2016) for methodology and construction algorithms surrounding phylogenetic networks. Note that to be able to account for autopolyploidization, we deviate from the usual notion of a phylogenetic network by allowing our phylogenetic networks to have parallel arcs (but no loops) – see e.g. Huber et al. (2021), Van Iersel et al. (2020) and the references therein for further results concerning such networks.

By taking for every leaf *x* of a binary phylogenetic network *N* on some finite set *X* the number of directed paths from the root of *N* to *x*, every phylogenetic network induces a multiplicity vector $\mathbf {m}$ indexed by the elements in *X*. Saying that *N* *realizes* $\mathbf {m}$ in this case (see Sect. 3 for an extension of this concept to phylogenetic networks) allows us to formalize our question as follows. Suppose $\mathbf {m}$ is a ploidy profile indexed by the elements of some finite set *X*. What can be said about the minimum number of hybrid vertices required by a binary phylogenetic network on *X* to realize $\mathbf {m}$? We call this number which is central to the paper the *hybrid number* of $\mathbf {m}$ and denote it by $h(\mathbf {m})$. If a binary phylogenetic network *N* has $h(\mathbf {m})$ hybrid vertices then we also say that *N* *attains* $\mathbf {m}$ (see again Sect. 3 for an extension of this concept to phylogenetic networks). The interested reader is referred to Steel (2016) for an overview of the related concept of the hybrid number of a set of phylogenetic trees (i.e. leaf-labelled rooted trees without any vertices of indegree and outdegree one whose leaf set is a pre-given set).

Before proceeding with presenting an example to help illustrate this question we remark that multiplicity vectors realized by binary phylogenetic networks have been used in Rossello et al. (2008) to define a metric for a certain class of binary phylogenetic networks. Furthermore, the stronger assumption that the number of directed paths from *every* vertex of a binary phylogenetic network *N* to every leaf of *N* is known, has led to the introduction of the concept of an ancestral profile for *N* Steel et al. (2019).

Returning to our question, consider the ploidy profile $\mathbf {m}=({12}, 6, 6, 5)$ indexed by $X=\{x_1,x_2,x_3,x_4\}$ where the multiplicity of $x_1$ is 12, that of $x_2$ and $x_3$ is 6, and that of $x_4$ is 5. Since no binary phylogenetic network on one leaf and two hybrid vertices can realize the ploidy profile $\mathbf {m}'=(5)$ because it has at most $2^2=4$ directed paths from the root to the leaf, it follows that a binary phylogenetic network that realizes $\mathbf {m}'$ and therefore also $\mathbf {m}$ must have at least three hybrid vertices. In fact, the subnetwork $N'$ in bold of the phylogenetic network depicted in Fig. 1i is the unique (subject to letting the arc *a* finish at a subdivision vertex of an outgoing or incoming arc of the hybrid vertex *h* or letting *a* start at a subdivision vertex of an outgoing or incoming arc of the vertex *t*) binary phylogenetic network that realizes $\mathbf {m}'$ and uses a minimum number of hybrid vertices. To be able to realize the ploidy profile (6, 5) and therefore also the ploidy profile $\mathbf {m}''=(6,6,5)$ at least four hybrid vertices are therefore needed. By counting directed paths from the root to each leaf of the phylogenetic network depicted in Fig. 1i with $x_1$, the hybrid vertex $h'$ above $x_1$, the two incoming arcs of $h'$, and the arc $(h',x_1)$ removed and any resulting vertices of indegree and outdegree one suppressed clearly realizes $\mathbf {m}''$. Calling that phylogenetic network $N''$ then, in a similar sense as $N'$, we also have that $N''$ is unique. To obtain a binary phylogenetic network from $N''$ that realizes $\mathbf {m}$ at least one further hybrid vertex is needed. Again by counting directed paths from the root to each leaf, it is easy to check that the binary phylogenetic network $N(\mathbf {m})$ depicted in Fig. 1i realizes $\mathbf {m}$ and postulates five hybrid vertices. As we shall see as a direct consequence of Theorem 2, $h(N)=5$. As a further consequence of that theorem, we obtain a closed formula for the hybrid number of a ploidy profile (Corollary 1).

The outline of the paper is as follows. In the next section, we present some relevant basic terminology and notation concerning phylogenetic networks. This also includes an unfold-operation for phylogenetic networks and a fold-up operation that generates phylogenetic networks, both of which were introduced originally in Huber and Moulton (2006). In Sect. 3, we extend the concept of attainment from binary phylogenetic networks to phylogenetic networks and study structural properties of phylogenetic networks that attain ploidy profile. As part of this, we introduce the two main concepts of the paper: a simple ploidy profile and an attainment of a ploidy profile. In Sect. 4, we associate two binary phylogenetic networks to a simple ploidy profile $\mathbf {m}$ which we denote by $D(\mathbf {m})$ and $B(\mathbf {m})$, respectively. As we shall see, the former is based on the prime factor decomposition of a positive integer *m* and the latter on a binary representation of *m*.

In Sect. 5, we associate a sequence $\sigma (\mathbf {m})$ to a ploidy profile $\mathbf {m}$ which we call the simplification sequence of $\mathbf {m}$ (Algorithm 1). As part of this, we also present some basic results concerning such sequences. This includes an infinite family of ploidy profiles that shows that such a sequence can grow exponentially large. Denoting the last element of the simplification sequence for $\mathbf {m}$ by $\mathbf {m}_t$, we then employ a traceback through $\sigma (\mathbf {m})$ to obtain the aforementioned binary phylogenetic network $N(\mathbf {m})$ from a binary phylogenetic network that attains $\mathbf {m}_t$ (Algorithm 2). Motivated by our partial results for binary phylogenetic networks that realize a simple ploidy profile summarized in Theorem 1, we provide an upper bound on the hybrid number $h(\mathbf {m})$ of a ploidy profile $\mathbf {m}$ for special cases of $\mathbf {m}$ (Proposition 2).

After collecting some preliminary results for $N(\mathbf {m})$ in Sect. 5, we establish in Sect. 6 that $N(\mathbf {m})$ attains $\mathbf {m}$ for a large class of ploidy profiles $\mathbf {m}$ (Theorem 2). In Sect. 7, we turn our attention to computing the hybrid number of the ploidy profile of a simplified version of the aforementioned Viola dataset from Marcussen et al. (2012). We conclude with Sect. 8 where we outline potential directions of further research.

## Preliminaries

**Fig. 2:**
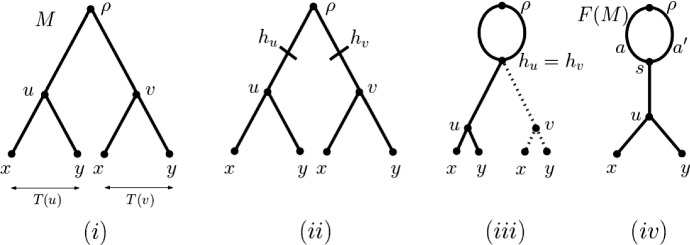
**i** The MUL-tree *M* obtained by unfolding the phylogenetic network on $X=\{x,y\}$ in **iv**. The trees *T*(*u*) and *T*(*v*) rooted at *u* and *v* and indicated with a double arrow, respectively, are equivalent. In fact, they are maximal inextendible. **ii** Subdivision of the incoming arcs of *u* and *v* by $h_u$ and $h_v$, respectively. **iii** Identifying the vertices $h_u$ and $h_v$. **iv** Deleting the subtree *T*(*v*) and the incoming arc of *v* (indicated by dotted lines in **iii**)

**Fig. 3:**
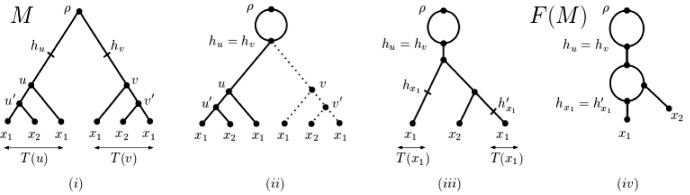
**i** The MUL-tree *M* obtained by unfolding the phylogenetic network on $\{x_1,x_2\}$ pictured in **iv**. The vertices *u* and *v* as indicated in **i** are the root of the maximal inexendible subtrees of *M* to which the subdivision, identification and deletion process described in Fig. 2 is applied to obtain the rooted directed acyclic graph *G* presented in **iii**. The two leaves labelled $x_1$ in *G* are the roots of two equivalent maximal inextendible subtree of *G* and applying the subdivision, identification, and deletion process to it results in *F*(*M*). In each case, the equivalent subMUL-trees are indicated by a double arrow

We start with introducing basic concepts surrounding phylogenetic networks. Subsequent to this, we briefly describe two basic operations concerning phylogenetic networks that are central for establishing a key result (Proposition 1). For the convenience of the reader, we illustrate both operations in Figs. 2 and 3 by means of an example. Throughout the paper we assume that *X* is a non-empty finite set. We denote the size of *X* by *n*.

### Basic concepts

Suppose for the following that *G* is a rooted directed connected acyclic graph which might contain parallel arcs but no loops. Then we denote the vertex set of *G* by *V*(*G*) and its set of arcs by *A*(*G*). We denote an arc $a\in A(G)$ starting at a vertex *u* and ending in a vertex *v* by (*u*, *v*) and refer to *u* as the *tail* of *a* and to *v* as the *head* of *a*. We call an arc $a\in A(G)$ a *cut-arc* if the deletion of *a* disconnects *G*. We call a cut-arc *a* of *G* *trivial* if the head of *a* is a leaf. Following Van Iersel et al. (2020), we call an induced subgraph of *G* with two vertices *u* and *v* and two parallel arcs form *u* to *v* a *bead* of *G*.

Suppose $v\in V(G)$. Then we refer to the number of arcs coming into *v* as the *indegree* of *v*, denoted by $indeg_G(v)$, and the number of outgoing arcs of *v* as the *outdegree* of *v*, denoted by $outdeg_G(v)$. If *G* is clear from the context then we will omit the subscript in $indeg_G(v)$ and $outdeg_G(v)$, respectively. We call *v* the *root* of *G*, denoted by $\rho _G$, if $indeg(v)=0$, and we call *v* a *leaf* of *G* if $indeg(v)=1$ and $outdeg(v)=0$. We denote the set of leaves of *G* by *L*(*G*). We call *v* a *tree vertex* if $outdeg(v)=2$ and $indeg(v)=1$. And we call *v* a *hybrid vertex* if $indeg(v)\ge 2$ and $outdeg(v)=1$. We denote the set of hybrid vertices of *G* by *H*(*G*). We call any two leaves *x* and *y* of *G* a *cherry*, denoted by $\{x,y\}$, if *x* and *y* share a parent. We say that *G* is *binary* if, $outdeg(\rho _G)=2$ and, for all $v\in V(G)-L(G)$ other than $\rho _G$, we have that the degree sum is three. We say that a vertex $w\in V(G)$ is *above* *v* if there exists a directed path *P* from *w* to *v*. In that case, we also say that *v* is *below* *w*. If, in addition, $v\not =w$ then we say that *w* is *strictly above* *v* and that *v* is *strictly below* *w*.

We call *G* a *(phylogenetic) network (on* *X*) if $L(G)=X$, every vertex $v\in V(G)-L(G)$ other than $\rho _G$ is a tree vertex or a hybrid vertex and $outdeg(\rho _G)= 2$. Note that phylogenetic networks in our sense were called semi-resolved phylogenetic networks in Huber and Moulton (2006). Also note that our definition of a phylogenetic network differs from the standard definition of such an object (see e.g. Steel 2016) by allowing beads. To emphasise that a phylogenetic network has no beads, we will sometimes refer to it as a *beadless* phylogenetic network.

Suppose *G* is a phylogenetic network on *X*. Then following Bordewich and Semple (2007), we define the *hybrid number* *h*(*G*) of *G* to be

$$\begin{aligned} h(G)=\sum _{h\in H(G)} (indeg(h)-1). \end{aligned}$$

We refer to a phylogenetic network *G* (on *X*) as a *phylogenetic tree (on X)* if $h(G)=0$. For a phylogenetic tree *T* on *X* and a non-root vertex $v\in V(T)$ we denote by *T*(*v*) the subtree of *T* obtained by deleting the incoming arc of *v* and the subsequently generated connected component that does not contain *v*.

Suppose that *N* is a phylogenetic network on *X*. Then we denote the number of directed paths from the root $\rho _N$ of *N* to a leaf *x* of *N* by $m_N(x)$. In case *N* is clear from the context, we will write *m*(*x*) rather than $m_N(x)$. For $N'$ a further phylogenetic network on *X*, we say that *N* and $N'$ are *equivalent* if there exists a graph isomorphism between *N* and $N'$ that is the identity on *X*. Furthermore, we say that $N'$ is a *(binary) resolution* of *N* if $N'$ is obtained from *N* by resolving all vertices in *H*(*N*) so that every vertex in $H(N')$ has indegree two. Note that for any resolution $N'$ of *N*, we have $h(N)=|H(N')|=h(N')$.

### The fold-up *F*(*U*(*N*)) of the unfold *U*(*N*) of a phylogenetic network *N*

Phylogenetic trees on *X* were generalized in Huber and Moulton (2006) to so called *multi-labelled trees (on X)* or *MUL-trees (on* *X*), for short, by replacing the leaf set of a phylogenetic tree by a multiset *Y* on *X*. Put differently, *X* is the set obtained from *Y* by ignoring the multiplicities of the elements in *Y*. As was pointed out in the same paper, every phylogenetic network *N* gives rise to a MUL-tree *U*(*N*) on *X* by recording, for every vertex *v* of *N*, every directed path from the root $\rho _N$ of *N* to *v*. More precisely, the vertex set of *U*(*N*) is, for all vertices $v\in V(N)$, the set of all directed paths *P* from $\rho _N$ to *v* where we identify *P* with its end vertex *v*. Two vertices *P* and $P'$ in *U*(*N*) are joined by an arc $(P',P)$ if there exists an arc $a\in A(N)$ such that *P* is obtained from $P'$ by extending $P'$ by the arc *a*. For example, the vertex *u* in Fig. 2i is the directed path $\rho$, *s*, *u* in the phylogenetic network in Fig. 2iv which crosses the arc *a*. The vertex *v* in Fig. 2i is the directed path $\rho$, *s*, *u* in Fig. 2iv which crosses the arc $a'$.

Reading Fig. 2 from left to right suggests that the unfolding operation can also be reversed. We next briefly outline this reversal operation which may be thought of as the fold-up of a MUL-tree *M* into a phylogenetic network *F*(*M*) (see Huber and Moulton 2006 for details, Huber et al. 2016; Huber and Scholz 2020 for more on both constructions, and Fig. 3 for an example). To make this more precise, we require further terminology. Suppose that *M* is a MUL-tree on *X*. Then we denote for a non-root vertex *v* of *M* the parent of *v* by ${\overline{v}}$. Extending the relevant notions from phylogenetic trees to MUL-trees, we say that a subMUL-tree *T* with root *u* of *M* is *inextendible* if there exists a subMUL-tree $T'$ of *M* with root vertex $w\not =u$ such that *T* and $T'$ are equivalent and either ${\overline{v}}={\overline{w}}$ or ${\overline{v}}\not ={\overline{w}}$ and $T({\overline{v}})$ and $T({\overline{w}})$ are not equivalent. By definition, every subMUL-tree of *M* that is equivalent with an inextendible subMUL-tree of *M* is necessarily also inextendible. In view of this, we refer to an inextendible subMUL-tree *T* of *M* as *maximal inextendible* if no subMUL-tree of *M* that is equivalent with *T* is a subMUL-tree of an inextendible subMUL-tree of *M*. So, for example, the subMUL-tree *T*(*u*) of the MUL-tree *M* depicted in Fig. 3i is inextendible but the subMUL-tree $T(u')$ is not. In fact, *T*(*u*) is maximal inextendible because the only equivalent copy of *T*(*u*) in *M* that is not *T*(*u*) is *T*(*v*) and neither *T*(*u*) nor *T*(*v*) is a subMUL-tree of an inextendible subMUL-tree in *M*.

To construct *F*(*M*), we first construct a sequence $\gamma _M$ of subMUL-trees of *M* which we call a *guide sequence* for *F*(*M*) and which we initialize with the empty sequence. Let *T* denote a maximal inextendible subMUL-tree of *M*. Let *u* denote the root of *T*, and let ${U=U_u}\subseteq V(M)$ denote the set of vertices $v\in V(M)$ such that the subMUL-tree rooted at *v* is equivalent with *T*(*u*). Note that, by definition, $|U|\ge 2$. Then, for all $v\in U$, we first subdivide the incoming arc of *v* by a vertex $h_v$ (cf Fig. 2ii and then identify all vertices $h_v$, $v\in U$, with the vertex $h_u$ (cf Fig. 2iii. By construction, $h_u$ clearly has |*U*| incoming arcs and also |*U*| outgoing arcs. From these |*U*| outgoing arcs of $h_u$, we delete all but one arc and, for each deleted arc *a*, we remove the subMULtree *T*(*v*) rooted at the head *v* of *a* (Fig. 2iv. We then grow $\gamma _M$ by adding an equivalent copy of *T*(*u*) at the end of $\gamma _M$ in case $\gamma _M$ is not the empty sequence. Otherwise we add *T*(*u*) as the first element to $\gamma _M$. Replacing *M* with the resulting graph $N_U$, we then find a new maximal inextendible subMUL-tree in $N_U$ and proceed as before (where we canonically extend the notions of a maximal inextendible subMUL-tree and of a subMUL-tree rooted at a vertex to $N_U$). In the case of the example in Fig. 3, the next maximal inextendible subMUL-tree in Fig. 3ii is one of the leaves labelled $x_1$.

By construction, the process of subdividing (cf Fig. 2ii, identifying (cf Fig. 2iii, and deleting (cf Fig. 2iv terminates in a phylogenetic network on *X*. That network is *F*(*M*). We depict *F*(*M*) in Fig. 3(iv) for the MUL-tree *M* pictured in Fig. 3i.

As was pointed out in (Huber and Moulton (2006), Section 6), *F*(*M*) is independent of the order in which ties are resolved when processing maximal inextendible subMUL-trees. Also, all tree vertices of *F*(*M*) have outdegree two because *M* is a binary MUL-tree. However, *F*(*M*) might contain hybrid vertices whose indegree is two or more since when processing a maximal inextendible subMUL-tree *T* there might be more than two subMUL-trees in the graph generated thus far that are equivalent with *T*. Finally, *F*(*M*) cannot contain arcs whose tail and head is a hybrid vertex because the hybrid vertices of *F*(*M*) are in bijective correspondence with the elements in the guide sequence for *F*(*M*).

We conclude the outline of both constructions with the following remark. Suppose *N* is a phylogenetic network on *X*. Then we call two tree vertices *u* and *v* in *V*(*N*) distinct an *identifiable pair* if the subMUL-trees of *U*(*N*) rooted at the vertex that is a directed path in *N* from the root $\rho _N$ of *N* to *u* is equivalent with the subMUL-trees of *U*(*N*) rooted at the vertex that is a directed path in *N* from $\rho _N$ to *v*. Let *C*(*N*) denote the *compressed* phylogenetic network obtained from *N* i. e. the phylogenetic network obtained from *N* by contracting all arcs (*u*, *v*) for which both *u* and *v* is a hybrid vertex. Bearing in mind that the phylogenetic network *F*(*M*) associated to a MUL-tree *M* was denoted ${\mathcal {D}}(M)$ in Huber and Moulton (2006), the following holds

1. *F*(*U*(*N*)) does not contain an identifiable pair of vertices (Huber and Moulton 2006, Theorem 3).
2. If *N* and $N'$ are phylogenetic networks such that the MUL-trees *U*(*N*) and $U(N')$ are equivalent then $h(F(U(N)))\le h(N')$ (Huber and Moulton 2006, Corollary 2(ii)).
3. If *N* is a phylogenetic network that does not contain an identifiable pair of vertices then the compressed phylogenetic networks $C(F(U(N)))=F(U(N))$ and *C*(*N*) are equivalent (Consequence of (R1) and (Huber and Moulton 2006, Theorem 2)).

## Properties of phylogenetic networks that attain the hybrid number of a ploidy profile

In this section, we collect structural properties of phylogenetic networks that attain the hybrid number of a ploidy profile. For ease of readability, we will assume from now on that for a ploidy profile $\mathbf {m}=(m_1,\ldots , m_n)$ on *X* the elements in *X* are always ordered in such a way that $m(x_i)=m_i$ holds for all $1\le i\le n$ and that $\mathbf {m}$ is in *descending order*, that is, $m_i\ge m_{i+1}$ holds for all $1\le i\le n-1$.

We start with some notations and definitions. Suppose that *N* is a phylogenetic network on $X=\{x_1,\ldots , x_n\}$ and that $\mathbf {m}=(m_1,\ldots , m_n)$ is a ploidy profile on *X*. Then we call $\mathbf {m}$ *simple* if $m_i = 1$ for all $2\le i\le n$ (i. e.  $m_1$ is the only component of $\mathbf {m}$ that is at least 2). Moreover, we call $\mathbf {m}$ *strictly simple* if $\mathbf {m}$ is simple and $|X|=1$. We say that *N* *realizes* a ploidy profile $\mathbf {m}$ if the elements in *X* can be ordered in such a way that $m_i=m(x_i)$ holds for all $1\le i\le n$. In this case, we also call *N* a *realization* of $\mathbf {m}$. Furthermore, we say that *N* is a *binary* realization of $\mathbf {m}$ if *N* is binary. We say that *N* *attains* $\mathbf {m}$ if *N* realizes $\mathbf {m}$ and $h(\mathbf {m})=h(N)=\sum _{h\in H(N)} (indeg(h)-1)$. In this case, we refer to *N* as an *attainment* of $\mathbf {m}$. If *N* is an attainment and also binary then we call *N* a *binary* attainment of $\mathbf {m}$.

**Fig. 4:**
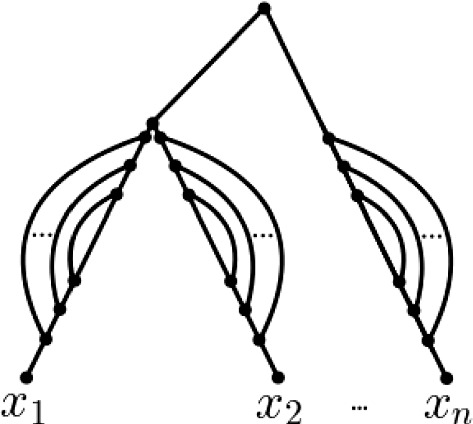
A phylogenetic network on $X=\{x_1,\ldots , x_n\}$ that realizes the ploidy profile $\mathbf {m}=(m_1,\ldots , m_n)$ on *X*. For all $1\le i\le n$, the number of curved lines is $m_i-1$

As is straight-forward to verify using the construction of the phylogenetic network indicated in Fig. 4 and the definition of *m*(*x*), $x\in X$, every ploidy profile $\mathbf {m}=(m_1,\ldots , m_n)$ on $X=\{x_1,\ldots , x_n\}$ with $n\ge 1$ is realized by a phylogenetic network that contains at most $\sum _{i=1}^n(m_i-1)$ hybrid vertices. Thus, the hybrid number of a ploidy profile always exists. As we shall see in Proposition 2, this bound can be improved for many ploidy profiles.

To be able to collect some simple properties of attainments which we will do next, we require further terminology and notation. Suppose *N* is a binary phylogenetic network on *X*. Then we say that *N* is *semi-stable* if *N* is equivalent to a resolution of *F*(*U*(*N*)). Motivated by the fact that a beadless phylogenetic network *N* that is equivalent to *F*(*U*(*N*)) was called *stable* in Huber et al. (2016), we canonically extend this concept to our types of phylogenetic networks by saying that a phylogenetic network *N* is *stable* if *N* is equivalent with *F*(*U*(*N*)).

**Fig. 5:**
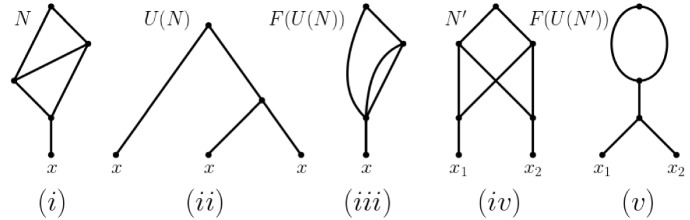
The phylogenetic network *N* depicted in **i** is semi-stable but not stable since it is not equivalent with *F*(*U*(*N*)) i. e. the phylogenetic network depicted in **iii**. the MUL-tree *U*(*N*) is pictured in **ii**. The phylogenetic network pictured in **iv** is not semi-stable. For a phylogenetic network to be stable it cannot contain the phylogenetic network $N'$ pictured in **iv** as an induced subgraph since $F(U(N'))$

For example, the binary phylogenetic network *N* depicted in Fig. 5i is semi-stable but not stable since *U*(*N*) is the MUL-tree depicted in Fig. 5ii and *F*(*U*(*N*)) is the phylogenetic network depicted in Fig. 5iii. The phylogenetic network $N'$ pictured in Fig. 5iv is not semi-stable. In fact, for a binary phylogenetic network *N* to be stable it cannot contain the phylogenetic network $N'$ pictured in Fig. 5iv as an induced subgraph (where $x_1$ and $x_2$ need not be leaves in $N'$) since $F(U(N'))$ is the phylogenetic network depicted in Fig. 5v. As we shall see below, certain types of binary phylogenetic networks called beaded trees are examples of stable phylogenetic networks. Although introduced in Van Iersel et al. (2020) in the context of a study of binary phylogenetic networks whose root have indegree one and not zero as in our case, the main feature of beaded trees is that a hybrid vertex must be contained in a bead. In view of this, we call a binary phylogenetic network *N* on *X* a *beaded tree* if *N* is either a phylogenetic tree on *X* or every hybrid vertex is contained in a bead (see e. g. Huber et al. 2021 for more on such graphs). Then since a beaded tree *N* cannot contain an identifiable pair of vertices, it follows by (R3) that the compressed phylogenetic networks *C*(*N*) and *F*(*U*(*N*)) are equivalent. Since *N* is a beaded tree and so does not contain arcs whose tail and head are hybrid vertices, it follows that *C*(*N*) is in fact *N*. Thus, *N* must be stable.

Suppose *N* is an attainment of a ploidy profile $\mathbf {m}$ on *X* that contains a cut-arc *a*. Then deleting *a* results in two connected components $N_1$ and $N_2$, one of which contains the root of *N*, say $N_1$, and the other is a phylogenetic network on $X-L(N_1)$. For $x\not \in L(N_1)$ we let $N_1^x$ denote the phylogenetic network on $L(N_1)\cup \{x\}$ obtained from $N_1$ by adding a pendant arc $a'$ to *tail*(*a*) and labelling the head of $a'$ by *x*. For any phylogenetic network *N* on *X*, we denote by $\mathbf {m}(N)$ the ploidy profile on *X* realized by *N*.

### Lemma 1

Suppose that *N* is an attainment of a ploidy profile $\mathbf {m}$ on *X*. Then the following holds.

(i) *F*(*U*(*N*)) and any resolution of *F*(*U*(*N*)) is an attainment of $\mathbf {m}$.
(ii) *N* is semi-stable.
(iii) Suppose *N* contains a cut-arc *a* and $N_1$ and $N_2$ are the connected components of *N* obtained by deleting *a*. If $\rho _N\in V(N_1)$ and $x\not \in L(N_1)$ then $N_1^x$ is an attainment of $\mathbf {m}(N_1^x)$ and $N_2$ is an attainment of $\mathbf {m}(N_2)$.

### Proof

(i): Clearly, *U*(*N*) is the unfold of *N* and also of *F*(*U*(*N*)). In view of (R2), we obtain $h(F(U(N)))\le h(N)$. Since *N* is a attainment of $\mathbf {m}$ and *F*(*U*(*N*)) realizes $\mathbf {m}$ it follows that $h(N)\le h(F(U(N)))$ must hold too. Thus, $h(F(U(N)))= h(N)$. Consequently, *F*(*U*(*N*)) is an attainment of $\mathbf {m}$. To see the remainder, suppose for contradiction that *F*(*U*(*N*)) has a resolution *D* that is not an attainment of $\mathbf {m}$. Then $h(D)=h(F(U(N)))<h(D)$; a contradiction.

(ii): Since *N* is an attainment of $\mathbf {m}$ it cannot contain a pair of identifiable vertices as otherwise $h(F(U(N)))< h(N)$ would hold which is impossible in view of Assertion (i). By (R3) it follows that the compressed networks *C*(*N*) and *C*(*F*(*U*(*N*))) are equivalent. Hence *N* must be a resolution of *F*(*U*(*N*)).

(iii): Since *a* is a cut-arc of *N* and therefore cannot have a head that is a hybrid vertex, we have $h(\mathbf {m})=h(\mathbf {m}(N_1^x))+ h(\mathbf {m}(N_2))$. Since every directed path from the root of *N* to a leaf of $N_2$ must cross *a* because *a* is a cut-arc of *N* it follows that $m_N(y)=m_{N_1^x}(x)\times m_{N_2}(y)$ holds for all $y\in L(N_2)$. This implies the statement. $\square$

The unfold and fold-up operations described in Sect. 2.2 lie at the heart of the proof of Proposition 1.

### Proposition 1

Suppose $\mathbf {m}$ is a ploidy profile on $X=\{x_1,\ldots , x_n\}$ and that *N* is an attainment of $\mathbf {m}$. Then there must exist a directed path *P* from the root of *F*(*U*(*N*)) to $x_1$ in *F*(*U*(*N*)) such that every hybrid vertex in *F*(*U*(*N*)) lies on *P*. If, in addition, *N* is stable then *P* must be a directed path in *N*.

### Proof

Put $\mathbf {m}=(m_1,\ldots , m_n)$. Suppose for contradiction that there exists no directed path from the root $\rho$ of *F*(*U*(*N*)) to $x_1$ in *F*(*U*(*N*)) that contains all hybrid vertices of *F*(*U*(*N*)). Then since *N* is an attainment of $\mathbf {m}$, Lemma 1 implies that *F*(*U*(*N*)) is also an attainment of $\mathbf {m}$. Consequently, $h(N)=h(F(U(N)))$. Let $\gamma _{U(N)}:T_1,T_2,\ldots , T_l$, some $l\ge 1$, denote a guide sequence for *F*(*U*(*N*)). Without loss of generality we may assume that $l\ge 2$ since otherwise *F*(*U*(*N*)) only contains one hybrid vertex and, so, the proposition holds. Then there must exist some $i\in \{2,\ldots , l\}$ such that $T_i$ is not a subMUL-tree of $T_{i-1}$ as otherwise all hybrid vertices of *F*(*U*(*N*)) would lie on a directed path from $\rho$ to $x_1$. Without loss of generality, we may assume that *i* is as small as possible with this property, i. e.  $T_{j+1}$ is a subMUL-tree of $T_j$, for all $1\le j\le i-2$.

Let *M* denote the MUL-tree obtained from *U*(*N*) as follows. For $j\in \{1,i\}$ let $t_j$ denote the number of equivalent copies of $T_j$ in *U*(*N*). Let $t=\min \{t_1,t_i\}$. Then $t\ge 2$. Choose *t* equivalent copies $R_1,\ldots , R_t$ of $T_i$ in *U*(*N*). For all $1\le j\le t$, delete the incoming arc of the root $r_j$ of $R_j$. Next choose *t* equivalent copies of $T_1$ in *U*(*N*) and, for all $1\le j\le t$, subdivide the incoming arc of the root of $T_j$ by a vertex $s_j$. Note that this is possible since $T_1$ is the first element in $\gamma _{U(N)}$ and so cannot be *U*(*N*). Last-but-not-least, add the arcs $(s_j,r_j)$, for all $1\le j\le t$. Since this might have resulted in arcs whose head is not contained in *X* and also vertices that have indegree one and outdegree one, we clean the resulting MUL-tree by removing the former and repeatedly suppressing the latter. Also we repeatedly identify the root with its unique child if this has rendered it a vertex with outdegree one.

By construction, *F*(*M*) is a phylogenetic network that realizes $\mathbf {m}$. Furthermore, $h(F(M))=h(F(U(N)))-(t-1)=h(N)-(t-1)<h(N)$ must hold since $t\ge 2$; a contradiction as *N* is an attainment of $\mathbf {m}$.

The remainder of the proposition is an immediate consequence because *N* and *F*(*U*(*N*)) are equivalent in this case. $\square$

Since, as mentioned above, beaded trees are stable phylogenetic networks the corresponding result for beaded trees in (Van Iersel et al. 2020, Lemma 13) is a consequence of Proposition 1 (once an incoming arc has been added to the root).

### Lemma 2

Suppose $\mathbf {m}=(m_1,\ldots , m_n)$ is a simple ploidy profile on *X* such that $m_1$ is a prime number. Then any cut-arc in an attainment of $\mathbf {m}$ must be trivial.

### Proof

Suppose *N* is an attainment of $\mathbf {m}$. Then the phylogenetic network $N'$ obtained from *N* by removing, for all $2\le i\le n$, the cut arcs ending in a leaf $x_i$ of *N* as well as the leaves $x_i$ (suppressing the resulting vertices of indegree one and outdegree one and also the root in case this has rendered it an outdegree one vertex) is a phylogenetic network on $X'=\{x_1\}$. Note that since none of the elements $x_i$ indexing $m_i$, $2\le i\le n$, contributes to *h*(*N*), we have $h(N)=h(N')$. Thus, $N'$ is an attainment of the ploidy profile $\mathbf {m_1}=(m_1)$. Put $m=m_1$ and $x=x_1$. If $m\in \{2,3\}$ then the lemma clearly holds since the only cut arc of $N'$ is the incoming arc of $x_1$ and therefore is trivial. So assume that $m\ge 4$.

Assume for contradiction that $N'$ has a non-trivial cut-arc *a*. Let $N_1$ and $N_2$ denote the connected components of $N'$ obtained by deleting *a*. Assume without loss of generality that the root of $N'$ is contained in $V(N_1)$. Let $y\not \in L(N_1)$. Then since for all leaves *z* in a phylogenetic network *M* the number of directed paths from the root of *M* to *z* is $m_M(z)$ it follows that $m=m_{N'}(x)=m_{N_1^y}(y)\times m_{N_2}(x)$. Since $1\not \in \{m_{N_1^y}(y), m_{N_2}(x)\}$ and *m* is prime this is impossible. $\square$

## Realizing simple ploidy profiles

**Fig. 6:**
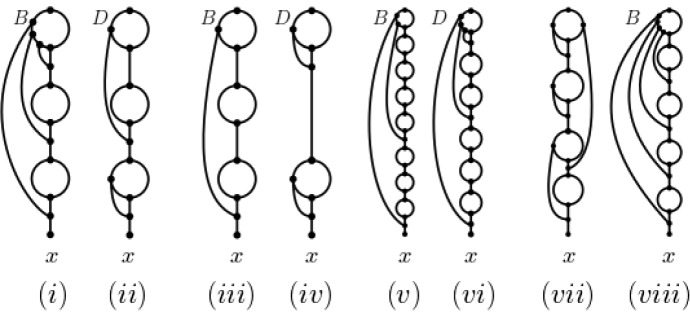
For a strictly simple ploidy profile $\mathbf {m}$ we depict in **i**, **iii**, **v** and **viii** the phylogenetic network $B=B(\mathbf {m})$ and in **ii**, **iv**, and **vi** the phylogenetic network $D=D(\mathbf {m})$. **i** and **ii**: $\mathbf {m}=(15)$ and $h(B)=6>5=h(D)$; **iii** and **iv**: $\mathbf {m}=(9)$ and $h(B)=4=h(D)$; **v** and **vi**: $\mathbf {m}=(265)$ and $h(B)=10<11=h(D)$. **vii** A realization of the ploidy profile $\mathbf {m}=(47)$ that uses eight hybrid vertices. **viii** The realization of the ploidy profile in **vii** in terms of $B(\mathbf {m})$

We start this section with associating to a simple ploidy profile $\mathbf {m}$ a binary phylogenetic network $D(\mathbf {m})$ that is based on the prime factor decomposition of $m_1$ and also a binary phylogenetic network $B(\mathbf {m})$ that is based on the unique bitwise representation of $m_1$. As we shall see, other ways to define binary realizations of $\mathbf {m}$ that are based on the prime factor decomposition of $m_1$ or on the bitwise representation of $m_1$ and that are similar in spirit to the definitions of $D(\mathbf {m})$ and $B(\mathbf {m})$ are conceivable. Furthermore, the ploidy profiles considered in Fig. 6 suggest that the relationship between the number of hybrid vertices in $D(\mathbf {m})$ and in $B(\mathbf {m})$ is not straight forward.

Suppose that $\mathbf {m}=(m_1,\ldots , m_n)$, $n\ge 1$, is a ploidy profiles on $X=\{x_1,\ldots , x_n\}$.

### The phylogenetic network $D(\mathbf {m})$

We begin with introducing further terminology. Suppose that *m* is a positive integer and that, for all $1\le i\le k$, $p_i$ is a prime and $\alpha _i\ge 1$ is an integer such that $p_1^{\alpha _1}p_2^{\alpha _2}\cdot \ldots \cdot p_k^{\alpha _k}$ is a prime factor decomposition of *m*. Without loss of generality, we may assume throughout the remainder of the paper that the primes $p_1,\ldots , p_k$ are indexed in such a way that $p_i>p_{i+1}$ holds for all $1\le i\le k-1$.

For all $1\le i\le k$, let $\mathbf {p}_i=(p_i)$ denote the strictly simple ploidy profile on $Y=\{x_1\}$. Also let ${\mathcal {A}}(\mathbf {p}_i)$ denote a binary phylogenetic network on *Y* that attains $\mathbf {p}_i$. Note that ${\mathcal {A}}(\mathbf {p}_i)$ need not be unique. For all $1\le i\le k$, we then define a binary phylogenetic network ${\mathcal {A}}(\mathbf {p}_i)^{\alpha _i}$ on *Y* as follows:

#### The phylogenetic network ${\mathcal {A}}(\mathbf {p}_i)^{\alpha _i}$

We take the root $\rho _i$ of ${\mathcal {A}}(\mathbf {p}_i)$ to be the root of ${\mathcal {A}}(\mathbf {p}_i)^{\alpha _i}$. If $\alpha _i=1$ then we take ${{\mathcal {A}}(\mathbf {p}_i)^{\alpha _i}}$ to be ${\mathcal {A}}(\mathbf {p}_i)$. If $\alpha _i\ge 2$ then we make $\alpha _i$ equivalent copies of ${\mathcal {A}}(\mathbf {p}_i)$ and order them in some way. Next, we identify the unique leaf of the first of the $\alpha _i$ copies of ${\mathcal {A}}(\mathbf {p}_i)$ under that ordering with the root of the second copy of ${\mathcal {A}}(\mathbf {p}_i)$ and so on until we have processed all $\alpha _i$ copies of ${\mathcal {A}}(\mathbf {p}_i)$ this way. The resulting directed acyclic graph is ${\mathcal {A}}(\mathbf {p}_i)^{\alpha _i}$ in this case.

To illustrate this construction, assume that $m=4$. Then $k=1$, $p_1=2=\alpha _1$, and $Y=\{x_1\}$. Furthermore, the phylogenetic network depicted in Fig. 3iv with the leaf $x_2$ and its incoming arc removed, and the resulting vertex of indegree and outdegree one suppressed, is ${\mathcal {A}}(\mathbf {p}_1)^{\alpha _1}$.

#### From ${\mathcal {A}}(\mathbf {p}_i)^{\alpha _i}$ to $D(\mathbf {m})$ in case $\mathbf {m}$ is strictly simple

Suppose $\mathbf {m}$ is strictly simple. Then we obtain $D(\mathbf {m})$ by ‘stacking’ the networks ${\mathcal {A}}(\mathbf {p}_1)^{\alpha _1},\ldots , {\mathcal {A}}(\mathbf {p}_k)^{\alpha _k}$ obtained as described above for a prime factor decomposition $p_1^{\alpha _1}p_2^{\alpha _2}\cdot \ldots \cdot p_k^{\alpha _k}$ of $m=m_1$ and a choice of attainment ${\mathcal {A}}(\mathbf {p}_i)$ of $\mathbf {p}_i=(p_i)$, for all $1\le i\le k$. If $k=1$ then $D(\mathbf {m})$ is ${\mathcal {A}}(\mathbf {p}_1)^{\alpha _1}$. So assume $k\ge 2$. Then we define $D(\mathbf {m})$ to be the phylogenetic network on $\{x_1\}$ obtained by identifying, for all $1\le i\le k-1$, the unique leaf of ${\mathcal {A}}(\mathbf {p}_i)^{\alpha _i}$ with the root of ${\mathcal {A}}(\mathbf {p}_{i+1})^{\alpha _{i+1}}$.

For the convenience of the reader, we depict $D(\mathbf {m})$ for the strictly simple ploidy profile $\mathbf {m}=(9)$ on $\{x\}$ in Fig. 6iv.

#### From ${\mathcal {A}}(\mathbf {p}_i)^{\alpha _i}$ to $D(\mathbf {m})$ in case $\mathbf {m}$ is not strictly simple

For all primes *p* in the prime factor decomposition of $m_1$, choose a binary attainment ${\mathcal {A}}(\mathbf {p})$ of the strictly simple ploidy profile $\mathbf {p}=(p)$ and construct the network $D(\mathbf {m'})$ for the strictly simple ploidy profile $\mathbf {m'}=(m_1)$ as described above. That network we then process further as follows. First, we choose an outgoing arc *a* of the root of $D(\mathbf {m'})$ and subdivide it with $n-1$ subdivision vertices $s_2, \ldots , s_n$ where, starting at the tail of *a*, the first subdivision vertex is $s_2$, the next is $s_3$, and so on. To the vertices $s_i$, $2\le i\le n$ we then add the arcs $(s_i,x_i)$ to obtain $D(\mathbf {m}$).

As an immediate consequence of the construction of $D(\mathbf {m})$, we have that $D(\mathbf {m})$ does not contain an identifiable pair of vertices. In view of (R1) it follows that $D(\mathbf {m})$ is semi-stable. In summary, we therefore have the following result.

##### Lemma 3

Suppose $\mathbf {m}$ is a simple ploidy profile on *X*. Then $D(\mathbf {m})$ is a binary, semi-stable phylogenetic network on *X* that realizes $\mathbf {m}$.

Note that as the strictly simple ploidy profile $\mathbf {m}=(m)$ with $m=265$ shows, the phylogenetic network depicted in Fig. 6v uses fewer hybrid vertices to attain $\mathbf {m}$ than the phylogenetic network $D(\mathbf {m})$ depicted in Fig. 6vi. Thus, an attainment of a simple ploidy profile $\mathbf {m}$ need not be obtained from a prime factor decomposition of the first component of $\mathbf {m}$.

For the reaminder of this section, assume again that $\mathbf {m}=(m_1,\ldots , m_n)$, $n\ge 1$ is a simple ploidy profile on $X=\{x_1,\ldots , x_n\}$.

### The phylogenetic network $B(\mathbf {m})$

We start with associating two vectors to a positive integer *m* which we call the *bitwise representation (of m)* and the *binary representation (of* *m*), respectively. For *m* a positive integer, the first is the 0-1 vector $\mathbf {v}_m= (v_m^f,\ldots , v_m^1, v_m^0)$ such that $m=\sum _{i=0}^f 2^i v_m^i$. For ease of presentation, and unless stated otherwise, we denote by $v_m^f$ the most significant bit that is one. The second is the vector $(i_1,\ldots , i_q)$, $q\ge 1$ and $i_j\not =0$, for all $1\le j\le q-1$, such that $m=\sum _{j=1}^q 2^{i_j}$ holds. Informally speaking, the *j*-th entry of that vector is the exponent of the term $2^{i_j}$ in the bitwise representation of *m*. Note that $2^{i_1}$ indexes the component $v_m^f$ of $\mathbf {v}_m$. For example, the bitwise representation of $m=11$ is (1, 0, 1, 1) and the binary representation of *m* is (3, 1, 0).

#### The phylogenetic network $B(\mathbf {m})$ in case $\mathbf {m}$ is strictly simple

Then $\mathbf {m}=(m_1)$ and $X=\{x_1\}$. Let *B*(*q*) denote the beaded tree with unique leaf $x_1$ and $q\ge 0$ hybrid vertices. Let $(i_1,\ldots ,i_q)$ denote the binary representation of $m_1$. Then $B(\mathbf {m})$ is obtained from the beaded tree $B(i_1)$ as follows. Choose one the two outgoing arcs of the root of $B(i_1)$ and subdivide it with $q-1$ vertices $s_2,\ldots , s_q$ not contained in $B(i_1)$ so that $s_2$ is the child of the root of $B(i_1)$, $s_3$ is the child of $s_2$, and so on. For all $1\le j\le q$, we then add an arc $a_j$ to $s_j$ whose head is a subdivision vertex of the outgoing arc of the hybrid vertex of $B(i_1)$ that has precisely $i_j$ hybridization vertices of $B(i_1)$ strictly below it.

We refer the interested reader to Fig. 6iii for an illustration of $B(\mathbf {m})$ for the strictly simple ploidy profile $\mathbf {m}=(9)$.

#### The phylogenetic network $B(\mathbf {m})$ in case $\mathbf {m}$ is not strictly simple

We first construct the phylogenetic network $B(\mathbf {m'})$ for the strictly simple ploidy profile $\mathbf {m'} =(m_1)$ on $\{x_1\}$. Next, we choose one of the two outgoing arcs of the root of $B(\mathbf {m'})$ and subdivide that arc with $n-1$ subdivision vertices $t_2,\ldots , t_n$ such that $t_2$ is the child of the root of $B(\mathbf {m'})$, $t_3$ is the child of $t_2$ and so on. Finally, we attach to each $t_i$ the arc $(t_i,x_i)$, $2\le i\le n$.

To illustrate this construction, consider the simple ploidy profile $\mathbf {m}_1=(5,1)$ on $X'=\{x_1,x_2\}$. Then $\mathbf {m'}=(5)$ and the phylogenetic network *D* depicted in Fig. 8 is $B(\mathbf {m})$. In fact, $B(\mathbf {m})$ is a binary attainment of $\mathbf {m}$.

As indicated in Fig. 6, the relationship between $D(\mathbf {m})$, $B(\mathbf {m})$, and a binary attainment of a simple ploidy profile $\mathbf {m}$ is far from clear in general. This holds even if $\mathbf {m}=(m)$ is strictly simple and *m* is a prime. Indeed for $m=47$ the hybrid number of $\mathbf {m}$ is at most eight since the phylogenetic network depicted in Fig. 6vi realizes $\mathbf {m}$. However $h(B(\mathbf {m}))=9$. This implies that, in general, $B(\mathbf {m})$ with $\mathbf {m}=(p)$ and *p* a prime cannot be used as an attainment with which to initialize the construction of $D(\mathbf {m})$.

As an immediate consequence of the construction of $B(\mathbf {m})$, we have the following companion result of Lemma 3 since similar arguments as in the case of $D(\mathbf {m})$ imply that $B(\mathbf {m})$ is semi-stable.

##### Lemma 4

Suppose $\mathbf {m}$ is a simple ploidy profile on *X*. Then $B(\mathbf {m})$ is a binary, semi-stable phylogenetic network on *X* that realizes $\mathbf {m}$.

To gain insight into the structure of $B(\mathbf {m})$, we next present formulae for counting, for a simple ploidy profile $\mathbf {m}$, the number $b(\mathbf {m})$ of vertices in $B(\mathbf {m})$ and also the number of hybrid vertices of $B(\mathbf {m})$. Note that such formulae are known for certain types of phylogenetic networks without beads (see e.g. McDiarmid et al. 2015; van Iersel and Kelk 2011 and Steel 2016 for more). To state them, we require further terminology. Suppose $m\ge 1$ is an integer and $\mathbf {v}_m$ is the bitwise representation of *m*. Then we denote by *p*(*m*) the number of non-zero bits in $\mathbf {v}_m$ bar the first one. For example, if $m=6$ then $p(m)=1$. Furthermore, we denote the dimension of a vector $\mathbf {v}$ by $\dim (\mathbf {v})$.

Armed with this, the construction of $B(\mathbf {m})$ from a simple ploidy profile $\mathbf {m}$ implies our first main result.

##### Theorem 1

Suppose that $\mathbf {m}=(m_1, m_2,\ldots , m_n)$, $n\ge 1$, is a simple ploidy profile. Let $\mathbf {i}_{m_1}=(i_1,i_2,\ldots , i_l)$, some $l\ge 1$, denote the binary representation of $m_1$. Then

$$\begin{aligned} {b(\mathbf {m})}=2({i_1+\dim (\mathbf {i}_{m_1})-1} + n-1) + 1{=2(\dim (\mathbf {v}_{m_1})-1+p(m_1)+n-1)+1} \end{aligned}$$

Furthermore, $B(\mathbf {m})$ has ${i_1+\dim (\mathbf {i}_{m_1})-1}$ hybrid vertices.

We remark in passing that in case $\mathbf {m}=(m)$ is strictly simple then any binary phylogenetic network *N* that realizes $\mathbf {m}$ has $2h(N)+1$ vertices since *N* has only one leaf and, so, the number of tree vertices of *N* plus the root must equal its number of hybrid vertices. Note that in case *N* is $B(\mathbf {m})$ then this also follows from Theorem 1 since $n=1$ and $i_1+\dim (\mathbf {i}_{m_1})-1$ is the number of hybrid vertices of *N* and therefore also the number of tree vertices of *N* plus the root.

## Realizing general ploidy profiles

**Fig. 7:**
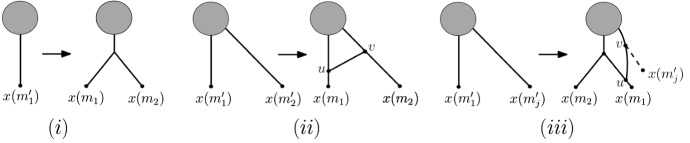
The three cases in the construction of the network $N(\mathbf {m})$ from a ploidy profile $\mathbf {m}=(m_1,m_2\ldots ,m_n)$ considered in Algorithm 2. For $\alpha =m_1-m_2$, the case $\alpha =0$ is depicted in **i**, the case $\alpha >m_2$ in **ii**, and the case $\alpha \le m_2$ in **iii**. In **iii**, the dashed arc and the vertex $x(m_j')$ are deleted and the vertex *v* is suppressed. In each case, the grey disk indicates the part of the phylogenetic network of no relevance to the discussion

**Fig. 8:**
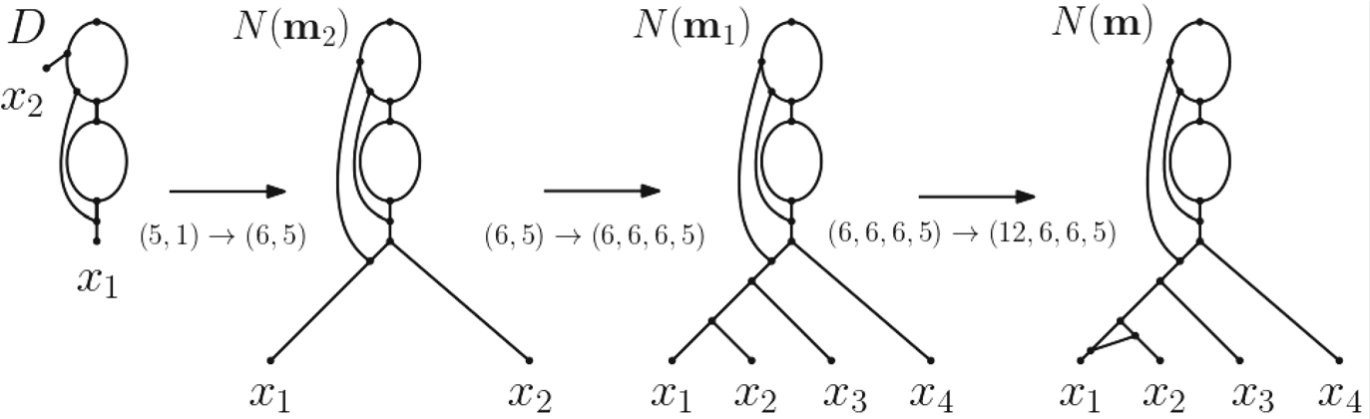
The construction of $N(\mathbf {m})$ for the ploidy profile $\mathbf {m}=(12,6,6,5)$ on $X=\{x_1,x_2, x_3, x_4\}$ where we have combined the steps $(6,5) \rightarrow (6,6,5)$ and $(6,6,5)\rightarrow (6,6,6,5)$ into the step $(6,5)\rightarrow (6,6,6,5)$. The leftmost network *D* on $X'=\{x_1,x_2\}$ is an attainment of $\mathbf {m}_t=(5,1)$ in the form of $B(\mathbf {m})$ and initializes the construction of $N(\mathbf {m})$. The network $N(\mathbf {m}_2)$ on $X'$ realizes the ploidy profile $\mathbf {m}_2=(6,5)$ and the network $N(\mathbf {m}_1)$ on *X* realizes the ploidy profile $\mathbf {m}_1=(6,6,6,5)$. The rightmost network is $N(\mathbf {m})$. The arrow labels indicate how a ploidy profile in $\sigma (\mathbf {m})$ was obtained

To help establish a formula for computing the hybrid number of a ploidy profile, we start by associating a binary phylogenetic network $N(\mathbf {m})$ on *X* to a ploidy profile $\mathbf {m}$ on *X* that realizes $\mathbf {m}$. This network is recursively obtained via a two-phase process which we present in the form of pseudo-code in Algorithms 1 (Phase I) and 2 (Phase II). We next outline both phases and refer the reader to Fig. 7 for an illustration of the three cases considered in Algorithm 2 and to Fig. 8 for an illustration of the construction of $N(\mathbf {m})$ from the ploidy profile $\mathbf {m}=(12,6,6,5)$. The phylogenetic network *D* in that figure is the phylogenetic network with which the construction of $N(\mathbf {m})$ is initialized.

Suppose $\mathbf {m}=(m_1,\ldots m_n)$ is a ploidy profile on *X*. Then, in Phase I, we iteratively generate a simple ploidy profile $\mathbf {m}_t$ from $\mathbf {m}$. This process is captured via a sequence $\sigma (\mathbf {m})$ of ploidy profiles which we call the *simplification sequence* for $\mathbf {m}$ and formally define as the output of Algorithm 1 when given $\mathbf {m}$ as input. The first element of $\sigma (\mathbf {m})$ is $\mathbf {m}$ and the last element is a simple ploidy profile which we call the *terminal element* of $\sigma (\mathbf {m})$ and denote by $\mathbf {m}_t$. We denote the number of elements of $\sigma (\mathbf {m})$ other than $\mathbf {m}$ by $s(\mathbf {m})$. Note that if $\mathbf {m}$ is a simple ploidy profile then $s(\mathbf {m})=0$ as $\mathbf {m}=\mathbf {m}_t$ holds in this case. Informally speaking, the purpose of $\sigma (\mathbf {m}): \mathbf {m}_0=\mathbf {m},\mathbf {m}_i,\ldots \mathbf {m}_{s(\mathbf {m})}=\mathbf {m}_t$ is to allow us to construct, for all $0\le i\le s(\mathbf {m})$, the network $N(\mathbf {m}_i)$ from $N(\mathbf {m}_{i+1})$ by reusing $N(\mathbf {m}_{i+1})$ (or parts of it) as much as possible (see Huber and Maher 2022 for more on such sequences).

To formally state Algorithm 1, we require further notations. Suppose $\mathbf {m}=(m_1,\ldots , m_n)$ is a ploidy profile on *X*. Then we denote for all $1\le i\le n$ the element of *X* that indexes $m_i$ by $x(m_i)$. Furthermore, for any non-empty sequence $\sigma$ and any *z*, we denote by $\sigma \cup \{z\}$ the sequence obtained by adding *z* to the end of $\sigma$.
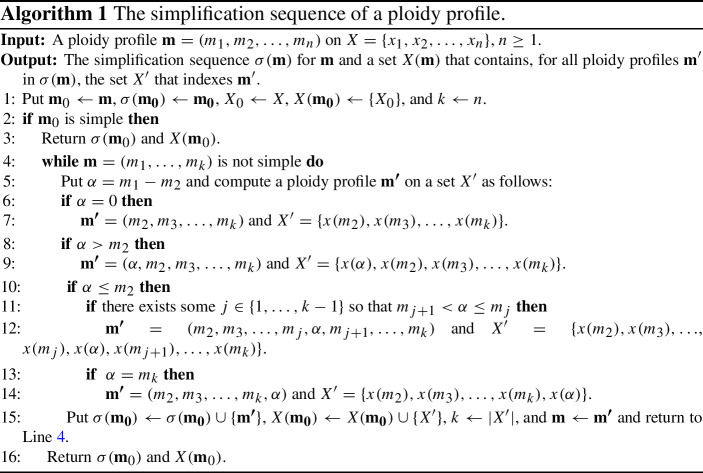


Phase II is concerned with generating the phylogenetic network $N(\mathbf {m})$ from the simplification sequence of $\mathbf {m}$ and the set $X(\mathbf {m})$ (for both see Phase I), and an attainment ${\mathcal {A}}(\mathbf {m}_t)$ of $\mathbf {m}_t$. Note that in case an attainment for $\mathbf {m}_t$ is not known, we can always initialize the construction of $N(\mathbf {m})$ with $D(\mathbf {m})$ or $B(\mathbf {m})$. The number of hybrid vertices of the generated network in this case is an upper bound on $h(N(\mathbf {m}))$ and therefore also on the hybrid number of $\mathbf {m}$.

To obtain $N(\mathbf {m})$, we use a trace-back through $\sigma (\mathbf {m})$ starting with $\mathbf {m}_t$. More precisely, assume that $\mathbf {m}_i=(m_1,\ldots , m_k)$, some $k\ge 2$ and $\mathbf {m}_{i+1}$ are two ploidy profiles in $\sigma (\mathbf {m})$, some $0\le i\le s(\mathbf {m})-1$. Then to obtain $N(\mathbf {m}_i)$ from $N(\mathbf {m}_{i+1})$ we distinguish again between the cases that $\alpha :=m_1-m_2=0$, $\alpha >m_2$ and $\alpha \le m_2$, see Fig. 7. Note that there might be non-equivalent attainments of $\mathbf {m}_t$ with which to initialize the construction of $N(\mathbf {m})$.
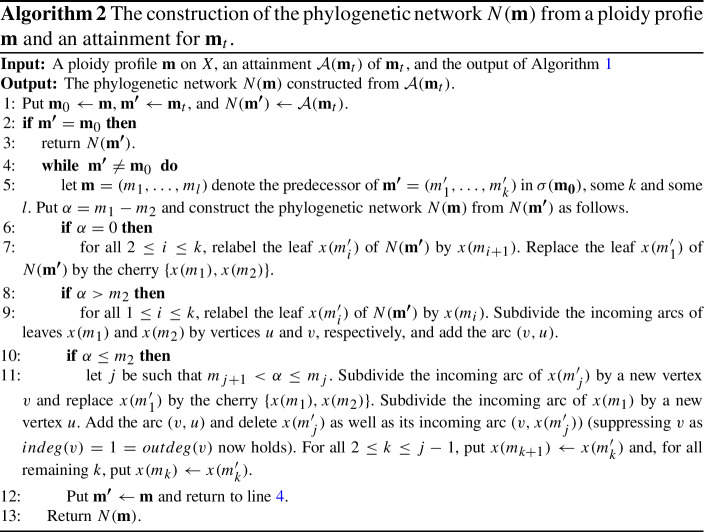

To illustrate the construction of $N(\mathbf {m})$, consider the ploidy profile $\mathbf {m}=(12,6,6,5)$ on $X=\{x_1,\ldots ,x_4\}$. Then $\mathbf {m}$, (6, 6, 6, 5), (6, 6, 5), (6, 5), (5, 1) is the simplification sequence $\sigma (\mathbf {m})$ associated to $\mathbf {m}$ because, by definition, the first element of $\sigma (\mathbf {m})$ is always $\mathbf {m}$. The ploidy profile (5, 1) is $\mathbf {m}_t$. The phylogenetic network *D* on $X=\{x_1,x_2\}$ on the left of Fig. 8 is an attainment of $\mathbf {m}_t$ in the form of $B(\mathbf {m}_t)$. Initializing Algorithm 2 with $B(\mathbf {m}_t)$ yields the phylogenetic network $N(\mathbf {m})$ at the right of that figure. Apart from the second arrow which is labelled $(6,5)\rightarrow (6,6,6,5)$ as it combines the steps $(6,5)\rightarrow (6,6,5)$ and $(6,6,5)\rightarrow (6,6,6,5)$, each arrow is labelled with the corresponding traceback step in $\sigma (\mathbf {m})$.

For any attainment ${\mathcal {A}}(\mathbf {m}_t)$ of the terminal element $\mathbf {m}_t$ of the simplification sequence $\sigma (\mathbf {m})$ of a ploidy profile $\mathbf {m}$ on *X*, the graph $N(\mathbf {m})$ is a phylogenetic network on *X* that realizes $\mathbf {m}$. Also, at each step in the traceback through $\sigma (\mathbf {m})$ the number of vertices is increased by exactly two. Denoting the number of vertices of $N(\mathbf {m})$ by $n(\mathbf {m})$ and the number of vertices in a binary attainment ${\mathcal {A}}(\mathbf {m}_t)$ of $\mathbf {m}_t$ by $a(\mathbf {m}_t)$, we obtain our next result.

### Lemma 5

Suppose $\mathbf {m}$ is a ploidy profile on *X*. Then for any binary attainment of $\mathbf {m}_t$ used in the initialization of the construction of $N(\mathbf {m})$, we have that $N(\mathbf {m})$ is a binary phylogenetic network on *X* that realizes $\mathbf {m}$. Furthermore, $n(\mathbf {m}) = a(\mathbf {m}_t) + 2 s(\mathbf {m})$.

In combination with Theorem 1, it follows that $N(\mathbf {m})$ has at most $b(\mathbf {m}_t) + 2 s(\mathbf {m})=2{(i_1+\dim (\mathbf {i}_{m_1})} + n +s(\mathbf {m}) +l)-3$ vertices and also at most ${ i_1+\dim (\mathbf {i}_{m_1})-1}+s(\mathbf {m})$ hybrid vertices where $\mathbf {m}_t=(m_1,\ldots , m_l)$, some $l\ge 1$, and $i_1$ is the first component in the binary representation $\mathbf {i}_{m_1}$ of $m_1$. Furthermore, we have

### Proposition 2

Suppose $\mathbf {m}=(m_1,\ldots , m_n)$ is a ploidy profile on *X* such that $B(\mathbf {m}_t)$ is a binary attainment of $\mathbf {m}_t$. For all $1\le k\le n$, let $(i_{k,1},\ldots ,i_{{k,l_k}})$ denote the binary representation of $m_k$, some $l_k\ge 1$. Then the following holds.

(i) $h(\mathbf {m})\le \sum _{k=1}^n ({i_{k,1}}+l_k-1)$. In case $\mathbf {m}$ is simple, $h(\mathbf {m})={i_{1,1}}+l_1-1$ which is sharp.
(ii) If $$m_i=2^{{i_{k,1}}} $$ holds for all $$1\le k\le n$$ then $$h(\mathbf {m})={i_{1,1}}$$.

### Proof

(i) To see the stated inequality, we construct a binary phylogenetic network *B* on $X=\{x_1,\ldots , x_n\}$ from $\mathbf {m}$ as follows. For all $1\le k\le n$, we first construct $B_k=B(\mathbf {m}_k)$ where $\mathbf {m}_k$ is the strictly simple ploidy profile $(m_k)$. Next, we add a new vertex $\rho$ and, for all $1\le k\le n$, an arc from $\rho$ to the root of $B_k$. If the resulting phylogenetic network on *X* is binary then that network is *B*. Otherwise, *B* is a phylogenetic network obtained by resolving $\rho$ so that $\rho$ has outdegree two.

By construction, *B* realizes $\mathbf {m}$ because $B_k$ realizes $\mathbf {m}_k$, for all $1\le k\le n$. By Theorem 1, it follows that $h(B_k)={i_{k,1}}+l_k-1$. Thus, $h(\mathbf {m})\le h(B)= \sum _{k=1}^n({i_{k,1}}+l_k-1)$, as required. If $\mathbf {m}$ is simple then $k=1$ and so $h(B)=h(B_1)={i_{1,1}}+l_1-1$.

(ii) This is a straight forward consequence of (i) and the fact that in this case $B_k$ is the beaded tree $B(i_{k,1})$. $\square$

Note that as the example of the ploidy profile $(k^l,k)$ for some $l,k\ge 2$ shows, there exists an infinite family of ploidy profiles $\mathbf {m}$ for which the length of the simplification sequence for $\mathbf {m}$ is at least $k^{l-1}+1$ and therefore grows exponentially in *l*. As a consequence of this, we also have, for any attainment of $\mathbf {m}_t$, that the number of hybrid vertices in $N(\mathbf {m})$ can grow exponentially in *l*. In view of this, we next study simplification sequences for special types of ploidy profiles. To this end we call an element $j\in \{1,\ldots , n\}$ *maximum* if $m_j$ is the last component of a ploidy profile $\mathbf {m}= (m_1,\ldots , m_n)$, $n\ge 1$, that is not one.

### Proposition 3

Suppose $\mathbf {m}=(m_1,\ldots , m_n)$ is a ploidy profile on *X*. Let *q* denote the maximum index of $\mathbf {m}$. Then the following holds

(i) If $k\ge 2$ is an integer such that $m_i=k$ holds for all $1\le i\le q$ then $s(\mathbf {m})=q-1$.
(ii) If $k\ge 1$ and $l\ge q+2$ are integers such that $m_i=k(l-i)$ holds for all $1\le i\le q$ then $s(\mathbf {m})={l+q-3}$.

### Proof

Note first that for both statements, we may assume without loss of generality that $q=n$ since elements in *X* with ploidy number one do not contribute to $s(\mathbf {m})$.

(i): Since $m_i=m_{i+1}$ holds for all $1\le i\le n-1$, the difference in dimension between any two consecutive ploidy profiles in $\sigma (\mathbf {m})$ is one. Hence, $q-1$ operations are needed to transform $\mathbf {m}$ into $\mathbf {m}_t$. Consequently, $s(\mathbf {m})=q-1$.

(ii): Since $m_{i-1}-m_i=k$ holds for all $2\le i\le q$, it follows that $q-1$ operations are needed to transform $\mathbf {m}$ into a ploidy profile $\mathbf {m'}$ of the form $(k(l-q), k,\ldots , k,1,\ldots , 1)$ where the components after the last *k* may or may not exist. To transform $\mathbf {m'}$ into a ploidy profile $\mathbf {m''}$ of the from $(k, k,\ldots , k,1,\ldots , 1)$ a further $l-q-1$ operations are needed. By Assertion (i), a further $q-1$ operations are needed to transform $\mathbf {m''}$ into a simple ploidy profile. Since $\sigma (\mathbf {m})$ is the concatenation of the underlying simplification sequences it follows that $s(\mathbf {m})=q-1+l-q-1+q-1=q+l-3$. $\square$

Together with Lemma 5, the next result may be viewed as the companion result of Lemmas 3 and 4 for general ploidy profiles.

### Proposition 4

For any ploidy profile $\mathbf {m}$ on *X* and any binary attainment of the terminal element in $\sigma (\mathbf {m})$, the graph $N(\mathbf {m})$ is a binary, semi-stable phylogenetic network on *X* that realizes $\mathbf {m}$.

### Proof

In view of Lemma 5, it suffices to show that $N(\mathbf {m})$ is semi-stable. Assume for contradiction that there exists a ploidy profile $\mathbf {m}=(m_1,\ldots , m_n)$ on *X* such that $N(\mathbf {m})$ is not semi-stable. Since the construction of $N(\mathbf {m})$ is initialized with an attainment of the terminal element $\mathbf {m}_t$ of $\sigma (\mathbf {m}): \mathbf {m}_0=\mathbf {m}, \mathbf {m}_1,\ldots , \mathbf {m}_l=\mathbf {m}_t$, some $l\ge 0$ and, by Lemma 1(ii), an attainment is semi-stable there must exist some $0\le i\le l$ such that the network $N(\mathbf {m}_i)$ is not semi-stable but all networks $N(\mathbf {m}_j)$, $i+1\le j\le l$ are semi-stable. Without loss of generality, we may assume that $i=0$. Put ${\mathbf {m'}=\mathbf {m}_1}$.

We claim first that $m_1\not =m_2$. Indeed, if $m_1=m_2$ then $\alpha =0$. Hence, Line 6 in Algorithm 2 is executed to obtain $N(\mathbf {m})$ from $N(\mathbf {m'})$. Since, by assumption, $N(\mathbf {m})$ is not semi-stable it follows that $N(\mathbf {m'})$ is not semi-stable; a contradiction. Thus, $m_1\not =m_2$, as claimed.

We next claim that $m_1>m_2$ cannot hold either. Assume for contradiction that $m_1>m_2$. Put $\alpha =m_1-m_2$. Assume first that $\alpha >m_2$. Then Line 8 in Algorithm 2 is executed to obtain $N(\mathbf {m})$ from $N(\mathbf {m'})$. Since $N(\mathbf {m'})$ is semi-stable, and this does not introduce an identifiable pair of vertices in $N(\mathbf {m})$, it follows that $N(\mathbf {m})$ is also semi-stable which is impossible.

So assume that $\alpha \le m_2$. Then Line 10 in Algorithm 2 is executed to obtain $N(\mathbf {m})$ from $N(\mathbf {m'})$. Similar arguments as in the previous two cases imply again a contradiction. This completes the proof of the claim.

Thus, $m_1<m_2$ must hold. Consequently, $\mathbf {m}$ is not a ploidy profile; a contradiction. Thus, $N(\mathbf {m})$ must be semi-stable. $\square$

## The hybrid number of a ploidy profile

In this section, we prove Theorem 2 which implies a closed formula for the hybrid number of a ploidy profile (Corollary 1). To help illustrate our theorem, we remark that for Line 8 in Algorithm 2 not to be executed we must have for every element $\mathbf {m'}=(m'_1,\ldots , m'_{n'})$, some $n'\ge 2$, in the simplification sequence of $\mathbf {m}$ that $m_1'>2m_2'$ does not hold.

### Theorem 2

Suppose $\mathbf {m}$ is a ploidy profile on *X* such that, for every ploidy profile in $\sigma (\mathbf {m})$, Line 8 in Algorithm 2 is not executed. If ${\mathcal {A}}(\mathbf {m}_t)$ is an attainment for $\mathbf {m}_t$ with which the construction of $N(\mathbf {m})$ is initialized then $N(\mathbf {m})$ is an attainment for $\mathbf {m}$.

### Proof

Put $\mathbf {m}=(m_1,\ldots , m_n)$ and assume that $\mathbf {m}$ is such that ${\mathcal {A}}(\mathbf {m}_t)$ is an attainment of $\mathbf {m}_t$. Suppose $X=\{x_1,\ldots , x_n\}$, $1\le n$. Note that we may assume that $n\ge 2$ as otherwise $\mathbf {m}$ is simple. Hence, $\mathbf {m}=\mathbf {m}_t$ and, so, the theorem follows by assumption on $\mathbf {m}_t$. Similar arguments as before imply that we may also assume that $\mathbf {m}$ is not simple.

Assume for contradiction that $N(\mathbf {m})$ is not an attainment of $\mathbf {m}$. Let *Q* denote an attainment of $\mathbf {m}$. Then $h(Q)<h(N(\mathbf {m}))$. In view of Proposition 1, there must exist a directed path *R* in *F*(*U*(*Q*)) from the root $\rho$ of *F*(*U*(*Q*)) to $x_1$ that contains all hybrid vertices of *F*(*U*(*Q*)). Since $h(Q)=h(F(U(Q)))$ as *C*(*Q*) and *F*(*U*(*Q*)) are equivalent by (R3), it follows that we may also assume that *Q* is binary and that *R* gives rise to a path *P* from $\rho$ to $x_1$ that contains all hybrid vertices of *Q*.

Since the construction of $N(\mathbf {m})$ is initialized with an attainment of $\mathbf {m}_t$, there must exist a ploidy profile $\overline{\mathbf {m}}$ in $\sigma (\mathbf {m})$ such that there exists a binary phylogenetic network ${\overline{Q}}$ that realizes $\overline{\mathbf {m}}$ and for which $h({\overline{Q}})<h(N(\overline{\mathbf {m}}))$ holds. Without loss of generality, we may assume that $\overline{\mathbf {m}}$ is such that for all ploidy profiles $\mathbf {m''}$ succeeding $\overline{\mathbf {m}}$ in $\sigma (\mathbf {m})$ we have $h(N(\mathbf {m''}))\le h(Q'')$ for all binary phylogenetic networks $Q''$ that realize $\mathbf {m''}$. For ease of presentation we may assume that $\overline{\mathbf {m}}=\mathbf {m}$.

Put $\mathbf {m'}=\mathbf {m}_1=(m_1',\ldots , m_{l'}')$, some $l'\ge 1$. Also, put $\alpha =m_1-m_2$, $N=N(\mathbf {m})$, and $N'=N(\mathbf {m}')$. Since Line 8 in Algorithm 2 is not executed for any element in $\sigma (\mathbf {m})$, it follows that either $\alpha =0$ or that $\alpha \le m_2$ since either Line 6 or Line 10 of that algorithm must be executed in a pass through the algorithm’s while loop.

**Case (a):** Assume that $\alpha =0$. Let $x_1=x(m_1)$ and $x_2=X(m_2)$ as in Line 7 in Algorithm 2. Let $2\le {r}\le n$ such that $m_1={m_r}$ holds. By the minimality of *h*(*Q*) it follows that the induced subgraph *T* of *Q* connecting the elements in $X_1=\{x_1,\ldots , {x_r}\}$ must be a phylogenetic tree on $X_1$ where, for all $3\le j \le k$, we put $x_j=x(m_j)$. Subject to potentially having to relabel the leaves of *T*, we may assume that $\{x_1,x_2\}$ is a cherry in *T*. Since $\alpha =0$ the directed acyclic graph $Q'$ obtained from *Q* by deleting $x_1$ and its incoming arc (suppressing resulting vertices of indegree and outdegree one) and renaming $x_{i+1}$ by $x(m_i')$, for all $1\le i\le n-1$, is a phylogenetic network on $\{x(m_1'),\ldots , x(m'_{n-1})\}$. Clearly, $Q'$ realizes $\mathbf {m'}$ since *Q* realizes $\mathbf {m}$. By assumption on $\mathbf {m}$ it follows that $N'$ is an attainment of $\mathbf {m'}$. Hence, $h(N')\le h(Q')$. Since *N* is obtained from $N'$ by executing Line 6 in Algorithm 2 it follows that $h(Q)<h(N)= {h(N')\le h(Q')}=h(Q)$ because *T* is a tree; a contradiction. Consequently, *N* must attain $\mathbf {m}$ in this case.

**Case (b):** Assume that $\alpha \le m_2$. Let *j*, $x_1$, and $x_2$ be as in Line 11 in Algorithm 2. We start with analyzing the structure of *Q* with regards to $x_1$ and $x_2$. To this end, note first that $m_2\ge 2$ must hold since otherwise $\mathbf {m}$ is simple and the theorem follows in view of our observation at the beginning of the proof.

By assumption on *Q*, there must exist a hybrid vertex *h* on *P* such that there is a directed path $P_h$ from *h* to $x_2$ because $m_2\ge 2$. Without loss of generality, we may assume that *h* is such that every vertex on $P_h$ other than *h* is either a tree vertex or a leaf of *Q*. Let *t* be the last vertex on *P* that is also contained in $P_h$.

**Fig. 9:**
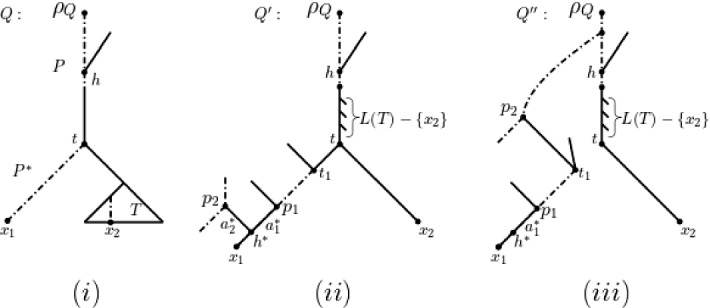
The transformation of *Q* **i** into the phylogenetic networks $Q'$ **ii** and $Q''$ **iii** as described in Case (b) of Theorem 2 for $p_1\not =p_2$. In each case, the dashed lines indicate paths. Note that in **iii** the dashed line could also start at $\rho _Q$

We next transform *Q* into a new phylogenetic network $Q''$ that is an attainment of $\mathbf {m'}$ (see Fig. 9 for an illustration). To do this, note first that since $m_2\not =m_1$ there must exist a hybrid vertex on *P* below *t*. We modify *Q* as follows to obtain a further attainment $Q'$ of $\mathbf {m}$. If *t* is the parent of $x_2$ then $Q'$ is *Q*. So assume that *t* is not the parent of $x_2$. Then we delete the subtree *T* of *Q* that is rooted at the child of *t* not contained in *P*. Note that *T* must have at least two leaves. Next, we subdivide the incoming arc of *t* by $|L(T)|-1$ subdivision vertices. To each created subdivision vertex we add an arc and bijectively label the heads of these arcs by the elements in $L(T)-\{x_2\}$. Next, we add an arc to *t* and label its head by $x_2$ so that *t* is now the parent of $x_2$. By construction, $Q'$ is a phylogenetic network on *X* that attains $\mathbf {m}$ because $h(Q)=h(Q')$.

Let $h^*$ be a hybrid vertex on the subpath $P^*$ of *P* from *t* to $x_1$ so that no vertex strictly below $h^*$ is a hybrid vertex of $Q'$. Let $a_1^*$ denote the incoming arc of $h^*$ that lies on $P^*$. Furthermore, let $a_2^*$ denote the incoming arc of $h^*$ that does not lie on $P^*$.

For $i=1,2$, let $p_i$ denote the tail of $a_i^*$. Note that $p_1=p_2$ might hold. Also note that the assumptions on *Q* imply that $p_1$ must be below *t*. Finally, note that $p_1$ must be a hybrid vertex unless $p_1=p_2$.

We claim that if $p_1\not =p_2$ then any vertex *v* on $P^*$ other than *t* and $x_1$ must be a hybrid vertex. Assume for contradiction that there exists a vertex $v\not \in \{t,x_1\}$ on $P^*$ that is a tree vertex. We show first that $p_2$ must also be below *t*. Since all hybrid vertices of *Q* lie on *P*, it follows that, *v* contributes at least $2m_2$ to the number of directed paths from $\rho$ to $x_1$ as $m_2$ is the number of directed paths from $\rho$ to $x_2$ and therefore, also from $\rho$ to *t*. Since $h_1^*$ contributes at least one further directed path from $\rho$ to $x_1$ in case $p_2$ is not below *t*, it follows that $m_1\ge \beta +{2}m_2$ for some $\beta \ge 1$. Hence, $m_2\ge \alpha =m_1-m_2 \ge \beta +{2}m_2-m_2\ge m_2$ because $\beta \ge 1$. Thus, $m_2=\beta +m_2$; a contradiction as $\beta \ge 1$. Hence, $p_2$ must also be below *t*, as required.

We next show that $p_2$ must be a vertex on $P^*$. Indeed, if $p_2$ were not a vertex of $P^*$ then it cannot be a hybrid vertex in view of our assumptions on *Q*. Thus, $p_2$ must be a tree vertex in this case. Since $p_1\not =p_2$ we obtain a contradiction as the choice of $h^*$ implies that $h^*$ is the parent of $x_1$. Thus, $p_2$ must be a vertex of $P^*$, as required. Since $p_2$ is a tree vertex it contributes at least $2m_2$ directed paths from $\rho$ to $x_1$. Since $p_1$ contributes at least a further $m_2$ directed paths from $\rho$ to $x_1$, we obtain a contradiction using similar arguments as before. Thus any vertex on $P^*$ other than *t* and $x_1$ must be a hybrid vertex in case $p_1\not =p_2$, as claimed.

We claim that if $p_1=p_2$ then $P^*$ has precisely 4 vertices and there exists two arcs from $p_1$ to $h^*$. To see this claim, note that $p_1$ contributes at least $2m_2$ directed paths from $\rho$ to $x_1$ because it is a tree vertex. If there existed a vertex *v* on $P^*$ distinct from $x_1$, $h^*$, $p_1$, *t* then *v* would contribute at least $m_2$ further directed paths from $\rho$ to $x_1$. Thus, we have again at least $3m_2$ directed paths from $\rho$ to $x_1$. Similar arguments as in the previous claim yield again a contradiction. By the choice of $h^*$ it follows that *t*, $p_1$, $h^*$ and $x_1$ are the only vertices on $P^*$. Since $p_1$ and $p_2$ are the parents of $h^*$ and $p_1=p_2$, it follows that there are two parallel arcs from $p_1$ to $h^*$. This concludes the proof of our second claim.

Bearing in mind the previous two claims, we next transform $Q'$ into a new phylogenetic network $Q''$ on *X* as follows. If $p_1\not =p_2$ then we first delete $a_2^*$ from $Q'$ and add an arc from $p_2$ to the child $t_1$ of *t* on $P^*$. Next, we remove the arc $(t,t_1)$ and suppress $h^*$ and *t* as they are now vertices with indegree one and outdegree one. The resulting directed acyclic graph is $Q''$. By construction, $Q''$ is clearly a phylogenetic network on *X*. Furthermore, the construction combined with our two claims, implies that $Q''$ realizes $\mathbf {m}'$ because the arc $(t,t_1)$ contributes $m_2$ directed paths from $\rho$ to $x_1$ in *Q* and therefore also in $Q'$. By construction, $h(Q'')=h(Q')-1=h(Q)-1$. Furthermore, $h(N)=h({N'})+1$ by the construction of *N* from $N'$. By the minimality of *h*(*Q*) and the choice of $\mathbf {m}$, it follows that $h(Q)<h(N)=h({N'})+1\le h(Q'')+1=h(Q)$; a contradiction. This concludes the proof of the theorem in case $p_1\not =p_2$.

If $p_1=p_2$ then we delete one of the two parallel arcs from $p_1$ to $h^*$ and suppress $p_1$ and $h^*$ as this has rendered them vertices of indegree one and outdegree one. The resulting directed acyclic graph is $Q''$ in this case. As before, $Q''$ is a phylogenetic network that, in view of our second claim, realizes $\mathbf {m'}$. Similar arguments as in the case that $p_1\not =p_2$ yield again a contradiction. This concludes the proof of the theorem in this case, and therefore, the proof of the theorem. $\square$

To illustrate Theorem 2, note that the ploidy profile $\mathbf {m}=(12, 6, 6, 5)$ in Fig. 1 satisfies the assumptions of Theorem 2. Consequently, the phylogenetic network $N(\mathbf {m})$ depicted in that figure is an attainment of $\mathbf {m}$.

**Fig. 10:**
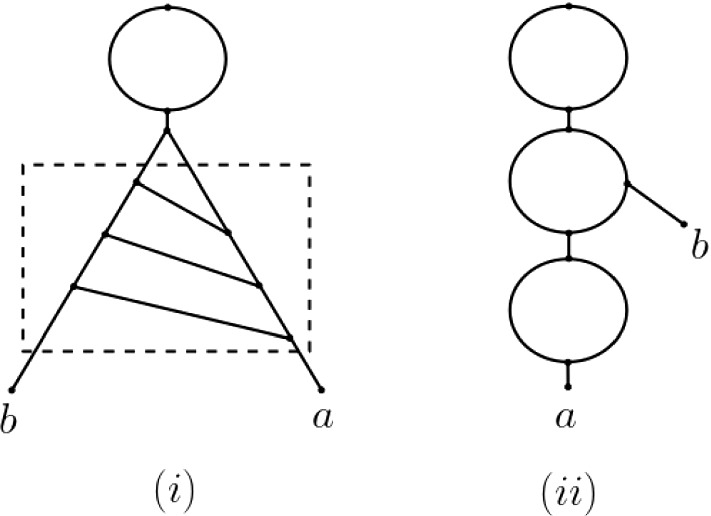
**i** The phylogenetic network $N(\mathbf {m})$ for the ploidy profile $\mathbf {m}=(8,2)$ on $X=\{a,b\}$ obtained via Algorithms 1 and 2. **ii** A phylogenetic network on *X* that attains $\mathbf {m}$ and has fewer hybrid vertices than $N(\mathbf {m})$

As the example depicted in Fig. 10 indicates, the assumption that Line 8 in Algorithm 2 is not executed is necessary for Theorem 2 to hold. In fact, if $\mathbf {m}$ is a ploidy profile such that $N(\mathbf {m})$ contains the subgraph highlighted by the dashed rectangle in the network in Fig. 10, then $N(\mathbf {m})$ can in general not be an attainment of $\mathbf {m}$.

**Fig. 11:**
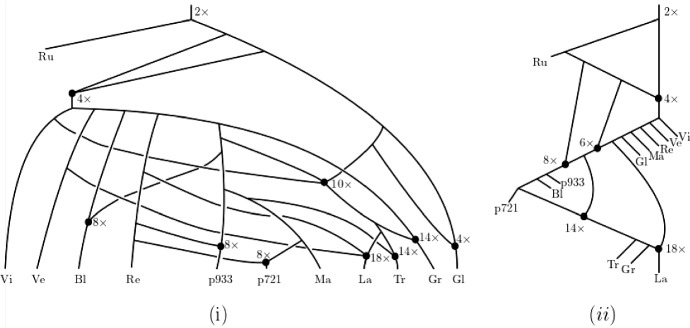
A phylogenetic network on leaf set $X = \{$ *V.langsdorffii, V.tracheliifolia, V.grahamii, V.721palustris, V.blanda, V.933palustris, V.glabella, V.macloskeyi, V.repens, V.verecunda,* Viola, Rubellium$\}$ adapted from a more general phylogenetic network that appeared as Figure 4 in Marcussen et al. (2012). Hybrid vertices are indicated with a filled circle and labelled by their corresponding ploidy number i. e. the number of directed paths from the root to the vertex times two because the root is assumed to be diploid. Leaves are labelled by the first two characters of their names (omitting *’V.’*, where applicable)

Theorem 2 and Case (b) in its proof combined with Theorem 1 and Proposition 2 implies our next result since $l-1$ additional hybrid vertices are inserted into $B(i_1)$ to obtain $B(\mathbf {m})$ where $\mathbf {m}$ is a simple ploidy profile and $(i_1,\ldots , i_l)$, $l\ge 1$, is the binary representation of the first component of $\mathbf {m}$. To state it we require a further definition. Let $\mathbf {m},\mathbf {m}_1,\ldots ,\mathbf {m}_i=(m_{1,i},\ldots , m_{p_i,i}),\ldots , \mathbf {m}_t$ denote the simplification sequence of a ploidy profile $\mathbf {m}$. Then we denote by $c(s(\mathbf {m}))$ the number of steps in $\sigma (\mathbf {m})$, for which $m_{1,i}>m_{2,i}$ holds where $0\le i\le s(\mathbf {m})$ and $p_i\ge 1$.

### Corollary 1

Suppose $\mathbf {m}$ is a ploidy profile such that Line 8 in Algorithm 1 is not executed when constructing $\sigma (\mathbf {m})$. Then $h(\mathbf {m})= h(\mathbf {m}_t)+c(s(\mathbf {m}))$. If $B(\mathbf {m}_t)$ is an attainment of $\mathbf {m}_t$ and $(i_1,\ldots ,i_l)$ is the binary representation of the first component of $\mathbf {m}_t$, some $l\ge 1$, then $h(\mathbf {m})= i_1+{l-1} +c(s(\mathbf {m}))$.

## A Viola dataset

In this section, we turn our attention to computing the hybrid number of the ploidy profile of a Viola dataset that appeared in more general form in Marcussen et al. (2012). Denoting that dataset by *X*, the authors of Marcussen et al. (2012) constructed a MUL-tree *M* on *X* and then used the PADRE software Huber et al. (2006) to derive a phylogenetic network *N* to help them shed light on the evolutionary past of their Viola species (Marcussen et al. 2012, Figure 4). We depict a simplified network $N'$ representing that past in Fig. 11i the only difference being that we have removed species that are not below a hybrid vertex of *N* as they do not contribute to the number of hybrid vertices of *N*. If more than one species were below a hybrid vertex of *N*, then we have also randomly removed all but one of them thereby ensuring that the hybrid vertex is still present in $N'$. The resulting simplified dataset comprises the taxa $x_1=$*V.langsdorffii*, $x_2=$*V.tracheliifolia*, $x_3$= *V.grahamii*, $x_4=$*V.721palustris*, $x_5=$*V.blanda*, $x_6=$*V.933palustris*, $x_7=$*V.glabella*, $x_8=$ *V.macloskeyi*, $x_9=$*V.repens* $x_{10}=$*V.verecunda*, $x_{11}=$*Viola*, and $x_{12}=$*Rubellium* (see Huber and Maher 2022 for more details on the simplified dataset). The labels of the internal vertices of $N'$ represent the ploidy number of the ancestral species represented by that vertex where we canonically extend the concept of a ploidy profile to the interior vertices of a phylogenetic network. By counting directed paths from the root to each leaf, it is easy to check, $h(N')=9$.

By taking directed paths from the root to the leaves of $N'$, we obtain the ploidy profile $\mathbf {m} = (9,7,7,4,4,4,2,2,2,2,2,1)$ on *X*. Note, since the root is diploid (labelled $2\times$), multiplying each component of $\mathbf {m}$ by two results in the ploidy numbers induced by the hybrid vertices in the network. The simplification sequence for $\mathbf {m}$ contains twelve elements and $\mathbf {m}_t=(2,1,1,1)$. Since an attainment of $\mathbf {m}_t$ must have one hybrid vertex and $D(\mathbf {m}_t)$ are equal $B(\mathbf {m}_t)$ and have one hybrid vertex each, it follows that $B (\mathbf {m}_t)$ is an attainment for $\mathbf {m}_t$. The phylogenetic network $N(\mathbf {m})$ obtained by initializing Algorithm 2 with $B(\mathbf {m}_t)$ is depicted in Fig. 11ii. Since at no stage in the construction of $N(\mathbf {m})$ Line 8 of that algorithm is executed, it follows by Theorem 2 that $N(\mathbf {m})$ is an attainment of $\mathbf {m}$. Counting again directed paths from the root to each leaf, it is easy to check that $N(\mathbf {m})$ has five hybrid vertices implying that $h(\mathbf {m})=5$. To compute the hybrid number of a ploidy profile whose components are not too large and, thererfore, we can find an attainment of its terminal element, we refer the interested reader to our R-function ‘ploidy profile hybrid number bound (PPHNB)’ which is obtainable from [1].

## Discussion

Motivated by the signal left behind by polyploidization, we have introduced and studied the problem of computing the hybrid number $h(\mathbf {m})$ of a ploidy profile $\mathbf {m}$. Our arguments apply, however, to any type of dataset that induces a multiplicity vector. Although stated within a phylogenetics context, the underlying optimization problem is, at its heart, a natural mathematical problem: “Given a multiplicity vector $\mathbf {m}$ find a rooted, leaf-labelled, directed acyclic graph *G* so that $\mathbf {m}$ is the path-multiplicity vector of *G* and the cyclomatic number of *G* is minimum”. Our results might therefore be also of relevance beyond phylogenetics.

Using the framework of a phylogenetic network, we provide a construction of a phylogenetic network $N(\mathbf {m})$ that is guaranteed to attain a ploidy profile $\mathbf {m}$ for a large class of ploidy profiles provided the construction of $N(\mathbf {m})$ is initialized with an attainment ${\mathcal {A}}(\mathbf {m}_t)$ of the terminal element $\mathbf {m}_t$ of the simplification sequence $\sigma (\mathbf {m})$ associated to $\mathbf {m}$. Members of that class include the ploidy profiles described in Proposition 3(ii). As a consequence, we obtain an exact formula for the hybrid number of $\mathbf {m}$ and also the size of the vertex set of $N(\mathbf {m})$ in terms of the length $s(\mathbf {m})$ of $\sigma (\mathbf {m})$ and the number $a(\mathbf {m}_t)$ of vertices of ${\mathcal {A}}(\mathbf {m}_t)$ for the members of our class. In case the ploidy numbers that make up $\mathbf {m}$ are not too large, both $c(s(\mathbf {m}))$ and $a(\mathbf {m}_t)$ can be computed easily by computing $\sigma (\mathbf {m}$) to obtain $c(s(\mathbf {m}))$ and using, for example, an exhaustive search for $a(\mathbf {m}_t$). Having said this, we also present an infinite family of ploidy profiles $\mathbf {m}$ for which $\sigma (\mathbf {m})$ grows exponentially. Motivated by this, we provide a bound for $h(\mathbf {m})$ and show that that bound is sharp for certain types of ploidy profiles. To help demonstrate the applicability of our approach, we compute the hybrid number of a simplified version of a Viola dataset that appeared in more general form in Marcussen et al. (2012). Our result suggests that the authors of Marcussen et al. (2012) potentially overestimate the number of polyploidization events that gave rise to their dataset.

Despite these encouraging results, numerous questions that might merit further research remain. These include “What can be said about $h(\mathbf {m})$ if the ploidy profile $\mathbf {m}$ is not a member of our class?”, and “Can we shed more light on the length of $\sigma (\mathbf {m})$ and also into attainments of the terminal element of $\sigma (\mathbf {m})$?”. Looking a little bit further afield, it might also be of interest to explore the relationship between so called accumulation phylogenies introduced in Baroni and Steel (2006) and ploidy profiles and also the relationship between ploidy profiles and ancestral profiles introduced in Steel et al. (2019).
